# Clinical Spectrum, Surgical Management, and Outcomes of *NR5A1*-Related 46,XY Differences of Sex Development: A Narrative Review

**DOI:** 10.3390/medicina61111965

**Published:** 2025-11-01

**Authors:** Stefania Vicario, Maria Escolino, Giorgia Esposito, Mauro Porcaro, Raffaella Di Mase, Mustafa Azizoglu, Ciro Esposito

**Affiliations:** 1 Division of Pediatric Surgery, Federico II University Hospital, 80131 Naples, Italy; vicstefy.94@gmail.com (S.V.); ciroespo@unina.it (C.E.); 2 Division of General Internal Medicine, Federico II University Hospital, 80131 Naples, Italy; 3 Division of Pediatric Radiology, Federico II University Hospital, 80131 Naples, Italy; 4 Division of Pediatric Endocrinology, Federico II University Hospital, 80131 Naples, Italy; 5 Department of Pediatric Surgery, Esenyurt Hospital, 34510 Istanbul, Turkey; mdmazizoglu@gmail.com; 6 Department of Stem Cell and Tissue Engineering, Istinye University, 34396 Istanbul, Turkey

**Keywords:** DSD, *NR5A1* gene, ambiguous genitalia, gonadal dysgenesis, tumor, gonadectomy

## Abstract

*Background and Objectives*: *NR5A1*-related 46,XY differences of sex development (DSD) represent a heterogeneous group of conditions characterized by variable degrees of undervirilization, gonadal dysgenesis, and endocrine dysfunction. Mutations in the *NR5A1* gene affect critical pathways of gonadal development and steroidogenesis, leading to complex diagnostic and management challenges. This narrative review aims to summarize the clinical spectrum, diagnostic algorithms, surgical management, and outcome data of pediatric *NR5A1*-related 46,XY DSD. *Materials and Methods*: A comprehensive search of PubMed, Scopus, and Web of Science databases was conducted, using terms related to *NR5A1* mutations, ambiguous genitalia, gonadal dysgenesis, tumor risk, and surgical management. A total of 26 studies were initially identified, of which 16 met the inclusion criteria for pediatric patients (≤18 years) with confirmed 46,XY karyotype, *NR5A1* mutation, and available clinical or surgical data. *Results*: *NR5A1* mutations are associated with phenotypes ranging from complete female external genitalia to apparently normal males with later infertility. While Sertoli cell function during fetal life is often preserved, Leydig cell dysfunction leads to incomplete masculinization. Spontaneous virilization during puberty has been reported. Management of gonadal dysgenesis remains controversial: while streak-like intra-abdominal gonads carry high germ cell tumor risk, warranting early gonadectomy, well-formed testes may be preserved under strict surveillance. *Conclusions*: *NR5A1*-related 46,XY DSD requires individualized, multidisciplinary management integrating genetic, endocrine, surgical, and psychosocial expertise. Gonadectomy decisions should be risk-stratified and, when possible, delayed to allow patients to participate in decision-making. Early psychological support and lifelong follow-up are essential to optimize physical and psychosocial outcomes.

## 1. Introduction

The definition of Differences of Sex Development (DSD) is generally used to describe a heterogeneous group of conditions characterized by discordance between chromosomal, gonadal, and phenotypic sex [1]. This topic remains a matter of debate about many aspects, including definition and classification.

Historically, the terms used to define such a condition were “intersexual”, “pseudohermaphroditism”, “hermaphroditism”, “sex inversion”. However, these terms were perceived as potentially dangerous because they only considered gender assignment, leaving out important elements such as the molecular genetic aspects of sexual development and respect for gender identity [2,3].

To integrate these aspects, the Consensus Statement of 2006 introduced a more modern and suitable nomenclature, which would not create controversy and confusion in the identification of pathological variants [3]. The main purpose was not to violate the rights to physical integrity, bodily autonomy, and self-determination, and the principle of free and informed consent [1,3].

One of the latest classifications proposed, after the Consensus update in 2016 [4], divided DSD into 3 categories: sex chromosome DSD, 46,XY DSD and 46,XX DSD. According to the 2025 EAU Guidelines on Paediatric Urology, DSDs are categorized into five major groups based on karyotype and pathophysiological mechanism: 46,XX DSD, 46,XY DSD, sex-chromosome DSD, ovotesticular DSD, and non-hormonal/non-chromosomal DSD [5] (Table 1). The 46,XX DSD group is characterized by female virilization, most commonly due to congenital adrenal hyperplasia (CAH). The 46,XY DSD group is heterogeneous, including conditions such as male undervirilization, gonadal dysgenesis, partial androgen insensitivity syndrome (PAIS), and complete androgen insensitivity syndrome (CAIS) [6]. Cases involving sex chromosome mosaicism —including 45,X; 45,X/46,XY; and 47,XXY karyotypes—may present with a normal male phenotype or asymmetric genital development. Ovotesticular DSD is defined by the presence of undescended gonads, ovotestes, and a wide phenotypic spectrum. Finally, non-hormonal and non-chromosomal DSD include conditions such as cloacal exstrophy, aphallia, and severe micropenis [4,5].

The main forms of 46,XY DSD are as follows: disorders of gonadal (testis) development, disorders of androgen synthesis or action, disorders of anti-Müllerian hormone (AMH) synthesis or action, and others [6]. All 46,XY DSD result from mutations in genes (e.g., *SRY*, *SOX*, *NR5A1*, *WT1*, *DHH*) involved in the embryological development of the genitalia. Mutations occurring at different stages of embryogenesis account for the heterogeneous clinical presentations observed. Genetic or environmental abnormalities may disrupt the embryogenetic process, leading to inadequate gonadal development with subsequent gonadal dysgenesis, as well as anomalies of the internal genitalia (absence or malformation) and external genitalia (insufficient or excessive virilization) [7,8,9].

The *NR5A1* gene, located on chromosome 9q33, encodes steroidogenic factor-1 (SF-1), a nuclear receptor that plays a pivotal role in adrenal and gonadal differentiation. The essential function of SF-1 was first demonstrated by Parker et al. in 1992, who showed that *NR5A1* knockout mice lacked both adrenal glands and gonads [10]. Subsequently, Achermann et al. (1999) identified the first human *NR5A1* loss-of-function variant in a 46,XY individual with gonadal dysgenesis and adrenal insufficiency, establishing the gene as a key determinant of human sex development [11]. Over the following two decades, additional studies expanded the clinical spectrum of *NR5A1* variants, showing that most affected patients have preserved adrenal function but widely variable degrees of gonadal dysgenesis, ranging from complete testicular failure to mild undervirilization [12,13].

Previous studies have mainly focused on genetic aspects and variant distribution of *NR5A1*-related DSD but provided limited information on the clinical and surgical management or long-term endocrine and psychosocial outcomes [12]. Moreover, single case-based narratives did not provide a structured synthesis for clinical pathways [13]. The complexity of this condition lies in the diagnostic challenges, gender assignment, surgical management, and long-term impact that each decision, including surgical therapy, will have on the child’s future quality of life. At present, no clear and universally accepted guidelines exist for the management of these patients, and the available data are limited and sometimes even contradictory, further complicating decision-making and standardization of care.

This narrative review specifically focuses on children with *NR5A1*-related 46,XY DSD, integrating genotype and clinical presentation with diagnostic algorithms, risk-stratified tumor surveillance, and indications, timing, and outcomes of gonadal surgery versus testis preservation, within a multidisciplinary, family-centered model.

## 2. Materials and Methods

A comprehensive search of the literature was conducted to identify all available studies reporting on pediatric patients with a 46,XY karyotype, ambiguous genitalia, and *NR5A1* gene mutations. The search was performed through PubMed, Scopus, and Web of Science databases, without restrictions on publication date or language.

The following search terms and Boolean combinations were applied: “DSD”, “differences of sex differentiation”, “*NR5A1* mutation”, “SF-1”, “ambiguous genitalia”, “non-palpable testes”, “tumor risk”, and “surgical management”. To broaden the search, the “related articles” function in PubMed was used, and the reference lists of all relevant studies were screened for additional eligible publications.

Inclusion criteria were pediatric patients (≤18 years of age) with a confirmed 46,XY karyotype; molecular diagnosis of *NR5A1* mutation (heterozygous or homozygous); clinical presentation with ambiguous genitalia or other signs of gonadal dysgenesis; and availability of data on genetic, clinical, diagnostic, or surgical aspects.

Exclusion criteria were patients with other genetic causes of DSD; studies exclusively involving adult or adolescent cohorts; and articles focused solely on genital reconstructive or cosmetic surgery without addressing genetic or endocrinological aspects.

All titles and abstracts were screened independently by two reviewers, and full texts were retrieved for potentially relevant studies. Discrepancies were resolved through discussion and consensus.

All available data on genetic aspects (type and location of mutation, inheritance pattern), clinical presentation (phenotype, associated anomalies, age at diagnosis), diagnostic workup (hormonal, radiological, and histopathological findings), management strategies (medical and surgical interventions), with particular attention to surgical indications, and outcomes (endocrine function, fertility potential, tumor risk, long-term follow-up) were extracted from the selected studies and analyzed. Given the heterogeneity of study designs and outcome measures, no formal meta-analysis was performed; instead, the evidence was summarized in the form of a narrative review focusing on diagnostic pathways, management strategies, and prognostic implications.

This study was exempt from Institutional Review Board (IRB) approval, as it was a narrative review of previously published data.

## 3. Results

From a total of 26 articles initially identified, 10 were excluded for various reasons: different gene mutations, different DSD subtypes, inclusion of adult or adolescent cohorts, or a primary focus on genital reconstructive/cosmetic surgery without genetic or endocrinological data. The selected articles included clinical studies, international guidelines, and retrospective analyses specifically addressing pediatric patients with *NR5A1*-related 46,XY DSD and ambiguous genitalia.

### 3.1. Steroidogenic Factor-1 (SF-1) and NR5A1 Gene

Steroidogenic factor-1 (SF1), encoded by the *NR5A1* gene (orphan nuclear receptor subfamily 5 group A member 1) located on the long arm of chromosome 9, is a key factor involved in gonadal embryogenesis, sex determination, and reproductive endocrine regulation and plays a pivotal role, especially in the context of 46,XY DSD [12,13,14,15,16].

These processes are governed by an intricate network of interacting genes, among which *NR5A1* functions as a transcription factor that modulates the expression of several genes, including *SRY*, *SOX9*, *GATA4*, *AMH*, *STAR*, and *CYP11A1* [17,18].

Gonadal development and sexual differentiation represent the final stages of a complex cascade initiated by the formation and stabilization of the primitive urogenital ridge during the undifferentiated phase of gonadal development [16].

At the early stage of 46,XY embryonic development, *NR5A1* is expressed in the urogenital ridge and contributes to the differentiation of the bipotential gonad into testes by modulating the transcription of *SRY*, *SOX9*, and *GATA4* [16,19].

In the sex determination stage, when the testis is developing, *NR5A1* upregulates the *SOX9* gene, a key regulator of testicular formation, promoting the differentiation of cell precursors in Sertoli cells [20]. Sertoli cells, in turn, induce the regression of Müllerian ducts through the secretion of AMH. *NR5A1* regulates other target genes, such as *SRY*, responsible for testicular differentiation, *WT1,* and *DHH*, associated with gonadal abnormalities and increased risk of tumor [16,20,21,22].

In the sex differentiation stage, the roles of *NR5A1* are: regulation of AMH and AMHR2 expression by Sertoli cells, promoting the transcription of steroidogenic genes, regulation of metabolic processes such as glycolysis, NADPH synthesis and colesterogenesis, and stimulation of the fetal Leydig cells to increase the expression of INLS3, STAR and LHCGR, necessary for testosterone synthesis and essential for virilization of both internal and external genitalia, descend of the testes and development of male reproductive system [23].

In the ovary, SF1 regulates steroid hormone biosynthesis in both granulosa and theca cells, only in the late stage of ovarian development, which depends on the absence of the Y chromosome, AMH, and testosterone production [24].

Mutations in *NR5A1* can impair normal sexual differentiation, accounting for 10–20% of 46,XY DSD cohorts [25]. These mutations can be responsible for steroidogenesis defects, due to altered production of adrenal and/or testicular hormones or altered peripheral response to steroid hormones, defined as hormone resistance syndrome, and complete or partial gonadal dysgenesis in patients with karyotype 46,XX or 46,XY, with a prevalence of 20% and 79%, respectively [16].

Mutations in the *NR5A1* gene are associated with a broad phenotypic spectrum affecting both 46,XY and 46,XX individuals. In 46,XY DSD, clinical manifestations include partial or complete gonadal dysgenesis, under-virilization with atypical external genitalia, hypospadias—with or without associated anomalies such as micropenis or bilateral anorchia—and spermatogenic failure. In 46,XX DSD, presentations may involve premature ovarian insufficiency (POI), 46,XX testicular DSD, or ovotesticular DSD. Pubertal and fertility-related features can include spontaneous virilization, normal or atypical pubertal progression, hypergonadotropic hypogonadism, amenorrhea with POI (in 46,XX), and oligo-azoospermia or spermatogenic failure (in 46,XY).

Mutations affecting the *NR5A1* gene can be frameshift, insertions, deletions, nonsense, complex variants, or more frequently missense variants, accounting for 58% of cases [26].

Although many of the heterozygous changes are De Novo (or even mosaic) events, it has been shown that approximately one-third of these changes are inherited in an autosomal dominant pattern, with maternal transmission [27]. Interestingly, in patients with heterozygous *NR5A1* variants, while steroidogenic function is impaired during fetal life, leading to alterations in testosterone levels, Sertoli cell function appears preserved during embryogenesis, as suggested by the absence of Müllerian remnants and the presence of primitive seminiferous tubules. However, Sertoli cell dysfunction tends to emerge during puberty, often reflected by elevated FSH levels [28,29]. This was described by Kostopoulou et al. in an adolescent patient, in whom increased FSH and decreased AMH concentrations indicated compromised Sertoli cell function during puberty, despite apparently normal activity during fetal development [29]. Furthermore, impaired testosterone production—observed after hCG stimulation or during puberty—suggests that *NR5A1*-related defects in steroidogenesis are more pronounced during fetal life than at later stages [29]. These findings support the hypothesis that *NR5A1* plays a more critical role in fetal steroidogenesis than in pubertal hormonal regulation [29].

In early development, *NR5A1* plays a key role in both the differentiation of Sertoli cells and the formation and/or maintenance of Leydig progenitor cells. However, the disruption of this pathway due to *NR5A1* mutations appears to predominantly affect fetal Leydig cells’ function, resulting in inadequate testosterone production and incomplete masculinization during the intrauterine period. In contrast, during puberty, the activation of the hypothalamic–pituitary–gonadal axis may trigger androgen production by adult-type Leydig cells, which are functionally more autonomous and less reliant on *NR5A1*, thus allowing delayed virilization [30,31].

The occurrence of spontaneous virilization despite impaired fetal masculinization suggests a dynamic, stage-dependent function of the steroidogenic pathway in individuals with *NR5A1* mutations. A widely accepted explanation lies in the developmental shift from fetal-type to adult-type Leydig cells. Adult Leydig cells, emerging during and after puberty, appear to be less dependent on *NR5A1*-mediated regulation for steroidogenesis. This may be partially compensated by the activity of *NR5A2*, a closely related nuclear receptor (also known as Liver Receptor Homolog-1, LRH-1), which may assume regulatory roles typically carried out by *NR5A1* in fetal life [28,32,33]. This decline in Sertoli cell function and the development of adult-type Leydig cells further support the concept that different testicular cell populations exhibit differential temporal sensitivity to *NR5A1* activity [31].

Mutations of the *NR5A1* gene can often determine other associated pathologies, including splenic and blood anomalies, endocrine anomalies, such as hypogonadism, bilateral anorchia or cryptorchidism, male infertility, ovarian failure, adrenal tumorigenesis, polycystic ovary syndrome, endometriosis, and anomalies of the nervous, skeletal, and cardiovascular system [16,34,35]. This is possible because this gene is expressed not only in the primary steroidogenic organs but also in non-steroidogenic tissues such as the brain, spleen, and even the placenta [34,36].

Adrenal involvement, although not always evident at birth, may appear later in life. Although *NR5A1* plays a role in the transcriptional regulation of key factors required for adrenal development, adrenal insufficiency is rarely associated with *NR5A1* mutations. Genetic events leading to *NR5A1* haploinsufficiency may impair testicular development, while adrenal function typically remains preserved into adulthood [27].

### 3.2. Epidemiology and Etiology

The incidence and prevalence of 46,XY DSD remain difficult to determine accurately due to the rarity of the condition, the heterogeneity of its clinical and genetic presentations, and the frequent underdiagnosis of milder forms [37,38]. Overall, DSDs are estimated to occur in approximately 1:4500–1:5500 live births, but specific subtypes such as partial or complete gonadal dysgenesis are significantly less common [37,38]. This epidemiological variability is also influenced by the fact that some cases, particularly those without clear genital ambiguity at birth, are only recognized later in life, often at puberty or during infertility investigations [6,34].

The etiology of 46,XY DSD is complex and multifactorial, involving genetic, biochemical, environmental, and epigenetic factors that can act individually or synergistically to interrupt the normal stages of sex determination and differentiation [34]. Monogenic causes include pathogenic variants in key sex-determining or sex-differentiating genes such as *SRY*, *SOX9*, *NR5A1*, *WT1*, and *DHH*, which are essential for gonadal development and hormonal regulation. Mutations in *NR5A1* account for 10–20% of 46,XY DSD cases, with both De Novo and inherited autosomal dominant transmission patterns, often showing variable penetrance [16,25,27]. Not all *NR5A1* variants cause overt, clinically significant DSD. Large cohort data showed variable expressivity, incomplete/sex-limited penetrance, presence of apparently unaffected carriers, and additional/oligogenic variants [25,26,27]. Oligogenic or polygenic origins have been increasingly recognized, potentially explaining more severe or atypical phenotypes [18,34].

Biochemical defects such as enzymatic deficiencies in steroidogenesis (e.g., 17β-hydroxysteroid dehydrogenase deficiency, 5α-reductase type 2 deficiency) or in AMH synthesis or action can result in incomplete regression of Müllerian structures or undervirilization [9,21,22]. Environmental factors include prenatal exposure to sex steroids or chemicals (e.g., pesticides, phthalates, bisphenol A), which have been shown in experimental and epidemiological studies to potentially interfere with the hormonal levels required for proper sexual differentiation, although their precise impact in humans remains debated [34]. Epigenetic mechanisms, including DNA methylation changes and chromatin remodeling, can alter the expression of sex-determining genes in the absence of structural mutations, representing an emerging field of research in DSD pathogenesis [34]. Ultimately, the interaction between these factors results in inadequate gonadal development, manifesting as partial or complete gonadal dysgenesis with abnormalities of the internal genitalia (hypoplasia, agenesis, or persistence of Müllerian structures) and external genitalia (either excessive or insufficient virilization). The phenotypic spectrum is wide, ranging from males with mild genital anomalies and infertility to those with marked genital ambiguity or a female phenotype, with or without virilization at puberty [6,16,34].

### 3.3. Clinical Presentation

The clinical presentation of neonates with DSD is highly variable and closely related to the specific mutation underlying the pathogenic mechanism [5,6]. Gonadal dysgenesis (GD) is defined as the incomplete or defective formation of the gonads, resulting from an abnormal process of germ cell migration and/or defective organization within the fetal gonadal ridge.

Patients with *NR5A1*-related 46,XY DSD may present at birth with a spectrum of phenotypes ranging from typical male genitalia with mild anomalies to complete female external genitalia. Common findings include ambiguous genitalia, micropenis, hypospadias, cryptorchidism, and bifid scrotum. In some cases, the phenotype may appear entirely female, with clitoral hypertrophy, posterior labial fusion, non-palpable gonads, and the presence of inguinal or labial masses. A thorough physical examination at birth is therefore essential.

In *NR5A1*-related 46,XY DSD, Sertoli cell function is often preserved during fetal life, as indicated by the absence of Müllerian structures, suggesting adequate AMH production in utero. However, Leydig cell dysfunction may result in insufficient testosterone production, leading to incomplete masculinization. Clinical expression is further complicated by the possibility of spontaneous virilization during puberty, despite a predominantly female or ambiguous phenotype at birth.

Regardless of genotype—which is often discordant—phenotypic distribution includes approximately 3% with ambiguous genitalia, 25% with apparent female phenotype, 16% with complete female phenotype, 17% with complete male phenotype, and 11% with apparent male phenotype [27]. An apparent female phenotype is defined by the presence of variable degrees of clitoral hypertrophy with non-palpable gonads, vulva with a single opening, posterior labial fusion, or an inguinal or labial mass. Conversely, an apparent male phenotype may present with bilateral non-palpable testes in a full-term apparently male infant, micropenis, severe penile curvature (for which length, width, and glans diameter should be measured), a single perineal orifice, severe hypospadias associated with bifid scrotum, or midshaft hypospadias associated with unilateral or bilateral undescended testis [4].

Although most cases of DSD are recognized in the neonatal period, some remain undetected at birth due to the absence of atypical external genitalia, leading to delayed diagnosis. In older children and young adults, physical findings may include genital or areolar hyperpigmentation, inguinal or labial mass in females, incomplete or delayed puberty, primary amenorrhea, virilization in individuals raised female, cyclic hematuria in males, and gynecomastia [4,6,7]. Hormonal profiles frequently reveal elevated follicle-stimulating hormone (FSH) and luteinizing hormone (LH) levels with low testosterone or its precursors, reflecting impaired testicular steroidogenesis.

The phenotypic variability of *NR5A1*-related 46,XY DSD has been further clarified by the largest international cohort study of individuals with *NR5A1*/SF-1 variants recently reported by Kouri et al. [36]. In this multicenter study including 197 individuals with *NR5A1*/SF-1 variants, 46,XY patients most frequently presented with a severe DSD phenotype (49%), while 23% showed an opposite-sex phenotype and 21% a typical male phenotype. Sex registration at birth varied, with approximately half of 46,XY individuals registered as female, and spontaneous pubertal virilization was reported in several cases. Of note, anomalies in other organ systems—particularly the spleen and, less frequently, the central nervous system—were identified in 27% of the cohort. These findings underline the remarkable clinical heterogeneity of *NR5A1*-related conditions and the importance of systematic phenotyping beyond gonadal and genital features.

One of the most challenging aspects in these cases is the possibility of spontaneous virilization during puberty, which has significant implications for sex assignment and management strategies, particularly when decisions are made in early childhood [16].

Given the unpredictability of pubertal outcomes, including potential spontaneous virilization and testicular hormonal activity, it is essential to closely monitor pubertal development in 46,XY patients with *NR5A1* mutations. This includes serial evaluation of androgen levels, gonadotropins, and AMH, along with imaging and clinical assessment. Such data are critical not only for individualized patient care but also for improving the understanding of long-term Leydig cell function and fertility potential in this population [33].

### 3.4. Diagnosis

The diagnosis of this rare disorder can be established at different temporal stages—prenatal, neonatal, or late-onset—through a range of diagnostic modalities that, although not yet standardized, require patient-specific adaptation followed by a multidisciplinary team of healthcare specialists [8].

During the prenatal period, non-invasive prenatal screening (NIPS) is performed in the first trimester, including genetic analyses to detect potential karyotypic abnormalities or determine fetal sex. A diagnosis is considered when discordance is identified between the results of these analyses and high-resolution second-trimester ultrasound demonstrating anatomical anomalies of the genitalia—indicating a genotype–phenotype mismatch—particularly in the context of a pertinent family history [37,39].

At present, prenatal diagnosis of *NR5A1*-related 46,XY DSD serves mainly diagnostic and counseling purposes rather than therapeutic intervention. No specific prenatal medical or surgical treatment is indicated. However, the identification of DSD during pregnancy enables multidisciplinary teams—including obstetricians, geneticists, neonatologists, and psychologists—to provide anticipatory guidance, support parental decision-making, and plan delivery in a tertiary care setting where specialized neonatal assessment and management are available immediately after birth [4,37].

When a DSD is suspected, a comprehensive family history should be obtained, including previous neonatal deaths, parental consanguinity, a family history of previous DSD or genital anomalies, primary amenorrhea or infertility in other family members, and maternal exposure to androgens during pregnancy [4].

After birth, the physical examination of patients with suspected DSD should include assessment of genital and areolar pigmentation, evaluation for hypospadias or the presence of urogenital sinus, and measurement of phallus size. Palpation is performed to determine the presence, position, and symmetry of the gonads. Blood pressure should also be measured, as certain endocrine disorders associated with DSD can present with hypertension or hypotension.

Within the first 48 h after birth, after physical examination confirming genital ambiguity, urine and blood analyses should be performed to evaluate urinary adrenal steroids, serum electrolytes, and the hormonal profile, including 17-hydroxyprogesterone (17-OHP), testosterone, dehydroepiandrosterone, gonadotropins, FSH, LH, cortisol, adrenocorticotropic hormone (ACTH), and AMH [15,40]. Hormonal studies are essential not only for diagnosis but also for monitoring and guiding potential hormone replacement therapy; therefore, they should be conducted in specialized laboratories with expertise in the interpretation of such results [1,3,37,40].

Patients with gonadal dysgenesis, 46,XY karyotype, and intra-abdominal gonads typically present with elevated LH and FSH levels, along with reduced concentrations of AMH, dihydrotestosterone (DHT), or testosterone precursors [37].

In the postnatal period, more detailed and comprehensive genetic investigations are performed to support gender assignment, guide appropriate management, and refine prognostic assessment. After polymerase chain reaction (PCR) amplification of the *SRY* gene—which enables rapid detection of the Y chromosome in a neonate—karyotype remains the most suitable investigation for classifying DSD. Additional analyses to identify sex chromosomes include quantitative fluorescent PCR, fluorescence in situ hybridization (FISH), array comparative genomic hybridization (aCGH), and single-nucleotide polymorphism (SNP) array. At present, in cases of 46,XY DSD karyotype, ambiguous genitalia and hormonal abnormalities without a well-defined genetic etiology, advanced genetic testing such as next-generation sequencing (NGS) and whole-exome sequencing (WES) can be performed, enabling a definitive genetic diagnosis in up to 50% of cases [16,41,42,43,44].

Regarding the radiological diagnosis, ultrasound (US) represents the first-line imaging modality. The reliability of prenatal US for determining fetal sex naturally depends on gestational age and the presence or absence of atypical genital development [39].

At birth, US of the abdominal, pelvic, inguinal, and perineal regions is useful to confirm or exclude the presence and localization of the gonads—whether intra-abdominal, inguinal, or labioscrotal—as well as to assess their consistency and morphology [45]. US is also necessary to identify possible Müllerian or utricular structures and the presence of the urogenital sinus. In selected cases, ultrasonographic evaluation of the urinary tract, adrenal glands, and terminal spine may be indicated to investigate associated conditions [3,46]. An HCG stimulation test may also be performed to confirm the presence of testicular tissue.

Although US is the preferred first-line investigation because it is easy to perform, requires neither sedation nor contrast administration, other radiological techniques are more suitable for precise diagnosis and detailed anatomical assessment [37]. These include magnetic resonance imaging (MRI), genitography for the assessment of vaginal duplication or urogenital sinus, genitoscopy, and cystoscopy, which, although more invasive, provide superior visualization of abnormal anatomical structures. In cases where intra-abdominal gonads with abnormal morphology are present and may raise suspicion of an ongoing fibrotic process, the most appropriate approach is to proceed with a diagnostic laparoscopy, which allows for gonadal biopsies and, when indicated, therapeutic intervention [4,37,47].

**Figure 1:**
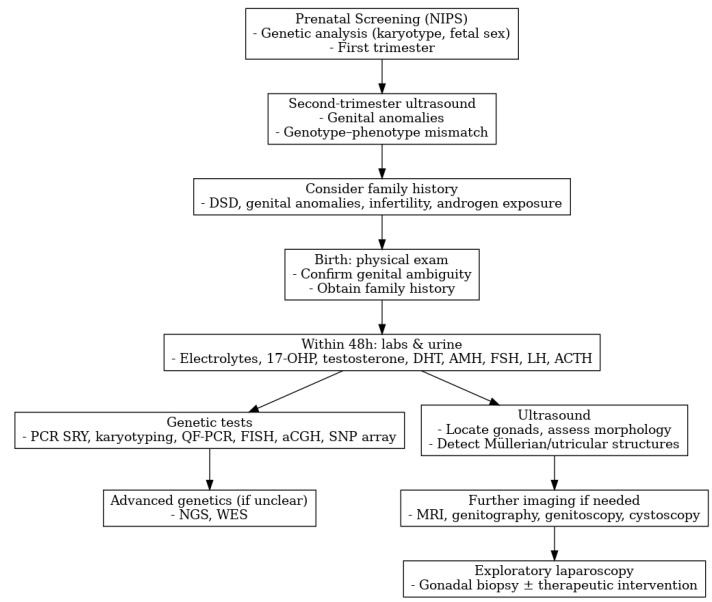
Diagnostic flowchart for DSD. Adapted from Lee PA et al. 2016 [4], and EAU Guidelines on Paediatric Urology 2025 [5].

Figure 1 summarizes the diagnostic flowchart for patients with DSD. This flowchart has been adapted from the 2016 International Consensus Statement and the 2025 EAU Guidelines on Paediatric Urology to provide a concise overview of the diagnostic approach to DSD in the pediatric population [4,5].

### 3.5. Gender Assignment

Gender assignment remains one of the most sensitive and debated aspects in the management of DSD patients, as it involves complex interactions between biological, psychological, social, and ethical factors [1,3]. In the neonatal period, this decision should primarily consider the choices of the newborn’s parents, who must be fully informed and supported by the multidisciplinary team—involving pediatric endocrinologists, urologists, surgeons, geneticists, psychologists, ethicists, and social workers—throughout the process.

Historically, gender assignment was mainly based on the appearance of external genitalia at birth, the presumed potential for surgical reconstruction, and prevailing cultural and religious practices. However, current practice incorporates a broader evaluation, including karyotype and genetic diagnosis, gonadal function and endocrine profile to assess the potential for spontaneous puberty and future fertility, tumor risk evaluation related to gonadal histology and chromosomal content, potential for sexual function based on genital anatomy and anticipated surgical outcomes [4].

In *NR5A1*-related 46,XY DSD, the decision-making process can be particularly complex because spontaneous virilization may occur during puberty despite a predominantly female or ambiguous phenotype at birth. This potential for delayed androgenization requires cautious initial gender assignment and reinforces the need to delay irreversible genital surgery until pubertal development can be anticipated or observed. Consequently, parental counseling should specifically address the dynamic endocrine course typical of *NR5A1* variants, which may modify gender identity outcomes over time.

In cases where there is uncertainty—particularly in newborns with ambiguous genitalia—current recommendations emphasize the importance of avoiding rushed decisions and postponing irreversible surgical interventions until sufficient diagnostic information is available and, when possible, the patient can actively participate in the decision-making. Temporary gender assignment may be used for administrative purposes, but this should be accompanied by clear communication to the family regarding the possibility of change if new clinical or genetic information emerges.

Psychological support is crucial for both the family and the patient, beginning from the time of diagnosis and continuing through childhood, adolescence, and adulthood. This support is crucial to assist in decision-making and prevent long-term psychosocial distress.

### 3.6. Multidisciplinary Team

**Figure 2:**
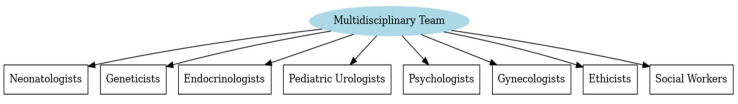
Multidisciplinary team for DSD management.

Patients with DSD require management by a multidisciplinary team, whose primary objective is to preserve both the physical and psychosocial well-being of the patient. The professionals involved include neonatologists, geneticists, endocrinologists, pediatric urologists, psychologists, gynecologists, ethicists, and social workers (Figure 2).

Each member of the team is responsible for maintaining effective communication and collaboration with the others to ensure that the management plan is individualized for each patient [48,49].

The aim is to provide parents/caregivers with comprehensive information regarding the patient’s condition, the available diagnostic and therapeutic options, the advantages, and disadvantages of each decision, and to guide them through every choice, especially the gender assignment [1,3]. This approach follows a shared care model allowing for the individualization of treatment while considering the substantial effects that DSD can have on mental health, quality of life, personal relationships, and sexual function in adult life [4,50].

In the context of *NR5A1*-related 46,XY DSD, the multidisciplinary team should also include experts with experience in rare gene-related DSD and long-term endocrine follow-up, as patients may exhibit evolving gonadal function, spontaneous pubertal changes, and variable fertility potential. Regular coordination among endocrinologists, geneticists, and surgeons is essential to adapt care strategies to these condition-specific developments.

### 3.7. Morbidity, Prognosis, and Cancer Risk

In patients with DSD, prognosis is influenced by multiple factors, including the type and timing of genital surgery, the need for lifelong hormone replacement therapy, the gonadal function, and, more importantly, the risk of malignant transformation [51,52].

Risk stratification schemes—such as those proposed by Looijenga et al. [53,54] and incorporated into the 2006 and 2016 international consensus statements [3,4]—classified DSD patients into high, intermediate, low, or no-risk categories. Besides complete and partial gonadal dysgenesis with Y chromosome material, other high-risk conditions include PAIS with non-scrotal testes, Frasier syndrome, and Denys–Drash syndrome. Intermediate-risk groups include Turner syndrome with Y chromosome material and certain enzymatic deficiencies (e.g., 17β-HSD). Low- or no-risk groups include ovotesticular DSD without Y material, complete androgen insensitivity syndrome (CAIS), 5α-reductase deficiency, and Leydig cell hyperplasia.

Among the various DSD subtypes, gonadal dysgenesis in individuals with a Y chromosome and intra-abdominal gonads is consistently classified as high risk for germ cell tumor (GCT) development, with an estimated malignancy risk of 15–35% according to the recent literature reports [3,52,53,54]. This elevated risk is primarily associated with the presence of the gonadoblastoma locus on the Y chromosome (GBY) and, more specifically, the testis-specific protein Y (*TSPY*) gene, now recognized as a key factor in tumorigenesis [55].

Histologically, premalignant lesions may present as germ cell neoplasia in situ (GCNIS) or gonadoblastoma containing undifferentiated gonadal tissue (UGT). The likelihood of progression to invasive GCT varies with the degree and pattern of gonadal differentiation, gonad location (intra-abdominal vs. scrotal), and patient age [53,54]. Cools et al. described five types of GCT occurring in the intersex gonad [54]. Type I includes (immature) teratoma/yolk sac tumor, presenting in gonadal and extragonadal midline sites in neonates/children, and originating from early primordial germ cells/gonocytes. Type II includes seminoma/nonseminoma of the testis (>15 years) and dysgerminoma/nonseminoma of the ovary or dysgenetic gonads (>4 years or congenital). Type III corresponds to spermatocytic seminoma of the testis in men >50 years, originating from spermatogonia/spermatocytes. Type IV includes ovarian dermoid cysts in all ages, originating from oogonia/oocytes, and type V includes hydatidiform moles of the placenta/uterus in the fertile period. Furthermore, they defined no risk for tumor development in patients with undervirilization and gonadal dysgenesis in the absence of germ cells or in the absence of the *TSPY* gene (in peripheral blood and/or gonadal tissue) [52,53,54,55].

Although management follows the general principles applied to DSD with Y-chromosome material, only isolated cases of germ-cell tumors have been reported in *NR5A1*-related 46,XY DSD [4,55]. Therefore, a risk-stratified approach is recommended, balancing gonadal preservation against tumor risk while considering the specific genotype and gonadal morphology in each patient. In high-risk intra-abdominal dysgenetic gonads, prophylactic gonadectomy should be considered, especially in phenotypic females or when histology reveals high-grade dysgenetic gonads or pre-neoplastic changes. For intermediate-risk gonads, close clinical and imaging surveillance is advised, with biopsy when indicated. For low- or no-risk cases, conservative management may be adopted, avoiding unnecessary surgical removal.

Ultimately, decisions must be individualized, balancing tumor risk against the benefits of gonadal preservation for endocrine function, fertility potential, and patient autonomy. Multidisciplinary follow-up is essential to ensure optimal long-term physical and psychological outcomes.

### 3.8. Surgical Management

In patients with gonadal dysgenesis, anatomical abnormalities are often associated with androgen resistance or defects in the biosynthesis of sex steroid hormones [1,2,3]. For this reason, the initial approach often involves medical therapy aimed at replacing the deficient hormonal profile—whether androgenic, estrogenic, or steroidogenic—tailored to the specific case. Parents should be actively involved in these decisions and fully informed about potential outcomes and possible complications [37,56].

Surgical treatment represents a controversial aspect. In patients with *NR5A1*-related 46,XY DSD, as in other forms of DSD, the decision to proceed with surgery must be considered cautiously [57]. Surgical options include gonadal surgery and external genital surgery—either for masculinization or feminization [1,3,4,58].

The decision regarding indications and timing of surgery should consider multiple factors, including the patient’s age, presence of a Y chromosome, specific DSD subtype, presence or absence of internal genitalia, and the position and hormonal function of the gonads [7,59]. In general, only necessary procedures that preserve function and avoid irreversible anatomical changes should be undertaken until the patient is able to provide clear and informed consent [1,3,4]. Consequently, reconstructive surgery for external genitalia to correct urogenital malformations—such as clitoral hypertrophy, hypospadias, micropenis, or penile curvature—should be deferred if functionality is not impaired. This approach allows the patient to participate actively in decision-making, preserving his/her physical and psychological well-being. Nonetheless, decisions should be individualized according to the phenotype and assigned gender, made by parents with appropriate support from a multidisciplinary team [3,4,37].

In childhood, surgical treatment is primarily focused on gonadal management. In *NR5A1*-related 46,XY DSD, the phenotype frequently includes intra-abdominal gonads, and the optimal surgical approach and timing remain controversial. The most debated aspect is whether to remove the gonads. Gonadectomy should be considered in cases of gonadal dysgenesis or dysplasia—considering the assigned gender— particularly when gonadal function is impaired and, most importantly, when there is a high risk of malignant transformation. Various malignancy risk classifications have been proposed over time, leading to different therapeutic approaches, without establishing definitive guidelines for management of *NR5A1*-related 46,XY DSD with intra-abdominal gonads [1,3,55].

Historically, prophylactic gonadectomy was recommended for all cases of gonadal dysgenesis with a 46,XY karyotype, *NR5A1* gene mutations, and intra-abdominal gonads, as these were considered high-risk for malignancy. Gonadectomy was particularly advised for non-scrotal gonads containing Y chromosomal material and for streak-like gonads with a fibrotic appearance [57].

According to the 2016 updated consensus statement [4], management strategies for gonadal dysgenesis (45,X/46,XY and 46,XY) differ according to the assigned gender. In males, undescended testes are typically treated with orchiopexy and biopsy, followed by regular surveillance. Self-examination is recommended, and an annual US should be performed after puberty. Post-pubertal biopsy is indicated, with decisions guided by US findings and previous biopsy results. If carcinoma in situ (CIS) is detected and progresses to gonadoblastoma (GB), gonadectomy is warranted. In cases of ambiguous genitalia, the threshold for gonadectomy is kept low to reduce malignancy risk.

In females, bilateral gonadectomy is generally performed at diagnosis to prevent GCT development. In patients with unclear gender assignment, gonadectomy is also considered at a low threshold when genital ambiguity is present, whereas in those with intact gonads, the decision is based largely on the patient’s confirmed gender identity.

The timing of gonadectomy remains controversial but primarily depends on the predicted malignancy risk. Since the risk is very low in the prepubertal period, the procedure can often be postponed, provided that the gonad can be safely monitored clinically or with US [60]. Recent evidence recommends primary gonadectomy for intra-abdominal dysgenetic gonads only if they are streak-like [4,5,7]. In other cases with increased GCT risk, non-palpable gonads should undergo laparoscopic exploration, repositioning to the lowest accessible site, and biopsy, followed by monitoring via palpation or US, with or without repeat biopsy. If lowering the gonad is not feasible, gonadectomy should be performed after multidisciplinary discussion and with informed consent from the parents and, when appropriate, the patient [4,5,7,55,61]. Gonadectomy is also indicated in cases of early GCT detection, anticipated adverse effects of sex steroid secretion, noncompliance with surveillance protocols (including self-examination and imaging), inaccessibility of the gonad to imaging, or patient preference.

**Figure 3:**
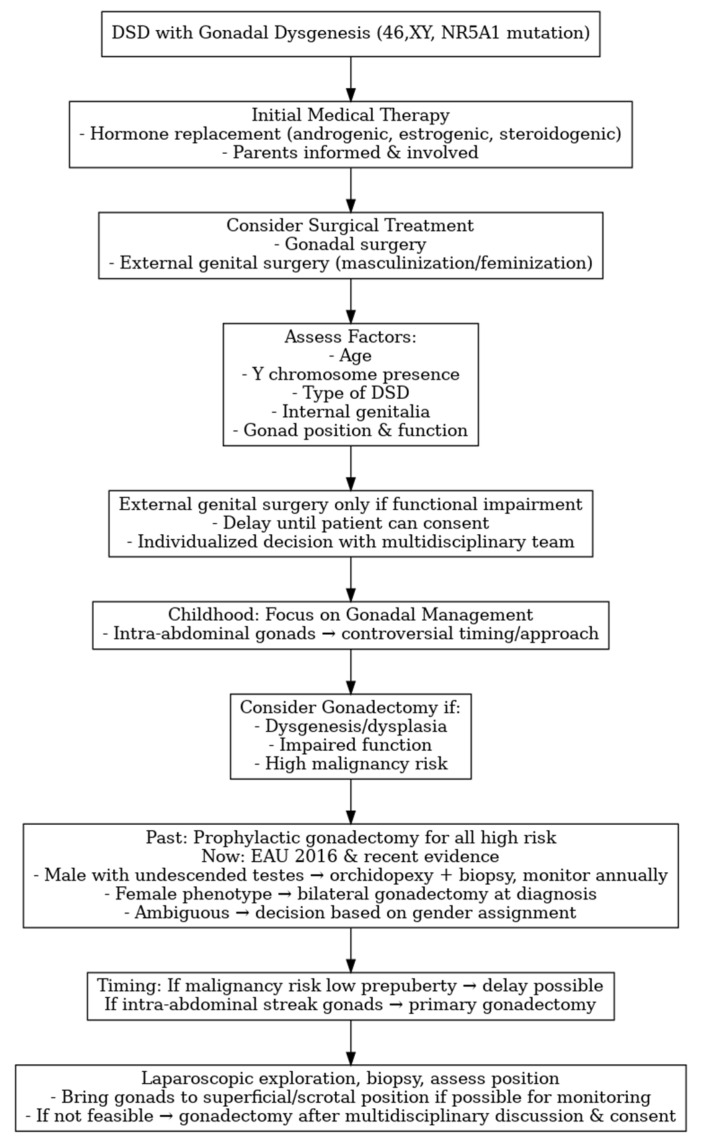
Management flowchart for *NR5A1*-related 46,XY DSD with gonadal dysgenesis.

Figure 3 summarizes the management flowchart for *NR5A1*-related 46,XY DSD with gonadal dysgenesis.

### 3.9. Quality of Life in Children and Adolescents

In pediatric populations, the quality of life (QoL) of individuals with DSD is often lower than that of their healthy peers, particularly in domains related to emotional well-being, social relationships, and school functioning [62,63,64]. Studies consistently report that psychiatric comorbidities are a major determinant of poorer QoL; children with DSD and a diagnosed psychiatric disorder score significantly lower on all PedsQL domains compared with those without such comorbidities [63,65]. Dissatisfaction with genital appearance, limitations in social participation, and heightened psychological distress are also common and may persist even after surgical or hormonal interventions [64,66].

Longitudinal evidence suggests that psychosocial functioning during childhood and adolescence strongly influences QoL in adulthood, with poorer early mental health correlating with lower adult QoL [66]. A significant reduction in QoL scores was reported in children’s self-reports, especially in self-esteem, physical well-being, and school functioning domains [67]. Cultural and healthcare access factors can further exacerbate these issues, as highlighted in low- and middle-income settings where late diagnosis and delayed interventions often result in more pronounced psychosocial difficulties [68]. Patients with visible physical differences or those who had undergone gender transition often faced social stigmatization. Such a situation was a significant source of stress, frequently leading to social isolation and withdrawal from interpersonal relationships. Promoting public education on DSD, stimulating self-esteem, and implementing timely medical interventions to limit atypical physical development may help reduce these barriers, thereby improving social acceptance and integration for affected individuals [69].

Overall, these findings emphasize the importance of early psychological assessment, timely medical management, and ongoing multidisciplinary support—including mental health professionals—to address the complex interplay between medical outcomes, psychosocial adaptation, and long-term QoL in children and adolescents with DSD.

### 3.10. Reproductive Potential in NR5A1-Related Conditions

- Inheritance and recurrence risk. Most *NR5A1* variants in affected families are heterozygous and behave as autosomal-dominant alleles with variable expressivity and incomplete penetrance; De Novo and (rarely) mosaic variants are also described [36]. Consequently, a heterozygous carrier has a 50% transmission risk to each child, but clinical outcomes range from severe 46,XY DSD to primary ovarian insufficiency (POI) in 46,XX or even apparently unaffected carriers, underscoring the need for individualized counseling.
- Fertility potential and options—46,XY. Spermatogenic impairment is frequent (from oligo- to azoospermia), but biological paternity is possible in selected cases, occasionally spontaneously and more often via assisted reproductive techniques (ART), including intracytoplasmic sperm injection (ICSI) with ejaculated sperm or surgically retrieved sperm (TESE/micro-TESE) when feasible. Early semen analysis and consideration of sperm banking are advisable in adolescents/young adults. If no viable sperm is available, donor sperm or adoption should be discussed. Given the 50% transmission risk and phenotype variability, couples should be offered Preimplantation Genetic Testing for Monogenic (PGT-M) or prenatal diagnosis when proceeding with biological reproduction [46].
- Fertility potential and options—46,XX. Many female carriers are asymptomatic, but a relevant proportion present with diminished ovarian reserve/POI, sometimes early [46]. Time-sensitive fertility preservation (oocyte or embryo cryopreservation) should be discussed before ovarian failure, and spontaneous pregnancies have been reported in some carriers. When ovarian reserve is depleted, oocyte donation is an effective option. As above, the inheritance risk is 50% with unpredictable expressivity in offspring (risk of 46,XY DSD in sons or POI in daughters).
- Shared decision-making. Current DSD consensus statements recommend that reproductive counseling accompany genetic diagnosis and long-term follow-up, integrating ART feasibility, PGT-M/prenatal testing, and psychosocial preferences within a multidisciplinary team (endocrinology, genetics, urology/gynecology, psychology) [4,5].

## 4. Discussion

*NR5A1*-related 46,XY DSD represents a clinically and genetically heterogeneous group of conditions that pose significant challenges in diagnosis, management, and long-term follow-up. The present review confirms that *NR5A1* gene mutations can manifest with a wide phenotypic spectrum, ranging from complete gonadal dysgenesis with female external genitalia to milder degrees of undervirilization or even apparently normal male genitalia with later infertility [14,16]. Recently, Kouri et al. [36] reported the largest international cohort of 197 individuals with *NR5A1*/SF-1 variants, confirming the wide phenotypic variability, lack of genotype–phenotype correlation, and the possible role of oligogenic inheritance in modulating disease expression. Interestingly, this study also highlighted the frequent presence of spleen anomalies in affected individuals and recommended systematic screening for these abnormalities. These findings further reinforce the concept that *NR5A1*-related 46,XY DSD represents a heterogeneous clinical entity influenced by multiple genetic and developmental factors rather than a purely monogenic disorder. This variability explains the diagnostic challenges encountered when managing these conditions, and highlights the complexity of genotype–phenotype correlations, which remain incompletely understood.

While some studies suggest that the position and nature of the *NR5A1* gene mutation influence clinical severity, overlapping phenotypes suggest the potential role of other genes and the contribution of environmental factors [17,18]. An interesting feature in *NR5A1*-related 46,XY DSD is the preservation of Sertoli cell function during fetal life, as evidenced by adequate AMH production and absence of Müllerian structures, despite impaired Leydig cell steroidogenesis leading to incomplete masculinization [26,29]. Interestingly, several reports have documented spontaneous virilization during puberty [31,33], potentially explained by the developmental transition from fetal to adult-type Leydig cells, which are less dependent on *NR5A1* for steroidogenesis and potentially supported by *NR5A2* activity [28,32]. This dynamic hormonal profile has significant implications for clinical management, particularly for early gender assignment, reinforcing the need to avoid irreversible surgical decisions in early childhood when the future pubertal development remains uncertain [4,37,38].

The diagnostic process for *NR5A1*-related DSD has been significantly improved with the advent of NGS [44], enabling earlier and more accurate identification of variants. Nevertheless, timely diagnosis remains challenging, especially in milder phenotypes without clear genital ambiguity at birth, leading to delayed diagnosis at puberty or during infertility evaluation [6,35]. Integrating genetic results with hormonal profiles, imaging findings, and family history is crucial to inform prognosis and guide multidisciplinary management [8,43]. Personalization of the diagnostic and therapeutic pathway is therefore essential, as it allows tailoring of care to the specific needs of each patient, maximizing functional and psychosocial outcomes.

The reported prevalence of *NR5A1* variants in 46,XY DSD (10–20% in specialized cohorts) should be interpreted in the context of major health-system and economic variability. Access to NGS—including targeted DSD gene panels and whole-exome sequencing—has markedly increased diagnostic yield and revealed that *NR5A1* variants are more frequent than initially believed. However, availability and reimbursement of molecular testing remain heterogeneous. In many countries, DSD diagnosis relies on referral to centralized multidisciplinary teams, but limited regional access and cost barriers can delay or prevent comprehensive genetic testing, leading to under-recognition of *NR5A1*-related conditions. According to the Society for Endocrinology (2016) and DSDnet/Endo-ERN recommendations, genetic diagnosis should be part of the first-line work-up for 46,XY DSD, since it guides clinical management, cancer-risk assessment, psychosocial support, and fertility counseling [1,46,70]. Broader NGS access and integration within national registries (e.g., I-DSD) have already demonstrated higher detection rates and improved care coordination [44,46]. Therefore, beyond individual patient benefit, promoting equitable access to genetic testing in DSD has public-health implications, enabling earlier diagnosis, informed decision-making, and more accurate family counseling across different healthcare settings.

From a surgical perspective, the management of gonadal dysgenesis in *NR5A1*-related 46,XY DSD remains controversial. Historically, prophylactic laparoscopic or open gonadectomy was recommended for all cases with streak-like, intra-abdominal, or dysgenetic gonads, given the increased risk of gonadoblastoma or other germ-cell neoplasms [54,57]. However, evidence regarding the actual incidence of malignancy in *NR5A1* variants remains limited, as only isolated cases of gonadal tumors have been reported [4,55]. In most reported *NR5A1*-related 46,XY cases, gonadectomy was performed due to dysgenetic gonads or increased risk of gonadal neoplasia [55,59]. Recent evidence from patients with CAIS has renewed interest in gonadal preservation as a safe, personalized option when stringent surveillance protocols are available [71]. Studies have shown that the absolute risk of gonadal malignancy in CAIS is relatively low during childhood and early adolescence, increasing mainly in adulthood. Therefore, many centers advocate a risk-stratified approach that defers or even avoids gonadectomy in selected cases, provided that regular clinical, hormonal, and radiological monitoring is ensured. Imaging—particularly MRI and high-resolution ultrasound—combined with serum tumor markers (LDH, hCG, AFP) and, when appropriate, immunohistochemical evaluation of OCT3/4 in gonadal biopsies, can help identify early neoplastic changes. This conservative strategy aims to preserve endogenous hormone production and potential fertility while minimizing surgical morbidity and the psychological impact of irreversible gonadectomy. In *NR5A1*-related 46,XY DSD, where the degree of gonadal dysgenesis and tumor risk varies according to the specific variant, a similar individualized approach may be warranted. The decision between prophylactic gonadectomy and active surveillance should thus be tailored to the patient’s genotype, gonadal morphology, endocrine function, and family preference, within the framework of a dedicated multidisciplinary DSD team [1,3,50].

Quality of life (QoL) in children and adolescents with DSD, including those with *NR5A1* mutations, is an increasingly recognized outcome measure. Several studies reported reduced QoL scores compared with healthy peers, particularly in emotional well-being, social relationships, and school functioning domains [62,63,64]. Psychiatric comorbidities, dissatisfaction with genital appearance, and limited social life participation and isolation are frequent determinants of poorer outcomes [65,66]. Importantly, early psychosocial support and family counseling have been shown to mitigate distress and improve adaptation, emphasizing the role of psychologists within DSD multidisciplinary teams along the entire diagnostic and therapeutic pathway [67]. Preserving both physical and psychological functions remains a core goal, ensuring not only survival and medical stability but also the best possible quality of life throughout the patient’s lifespan.

Given the lifelong implications of *NR5A1*-related DSD, follow-up should extend into adulthood to monitor endocrine status, reproductive health, tumor risk, and psychosocial well-being. Coordination between pediatric and adult services is critical to ensure continuity of care, particularly during transition phases [4,50].

This review has several limitations. First, the available literature on *NR5A1*-related 46,XY DSD in the pediatric population is limited, with most studies consisting of small case series or single case reports, which reduces the generalizability of findings. Second, the included studies are predominantly retrospective and heterogeneous in terms of study design, patient selection, and outcome measures, precluding direct comparison and quantitative analysis of data. Third, variability in diagnostic criteria, management protocols, and follow-up duration further complicates the interpretation of results. Additionally, the low number of longitudinal studies limits understanding of long-term outcomes, including fertility potential, endocrine function, and psychosocial adaptation.

Future research should be focused on establishing more robust genotype–phenotype correlations, clarifying the natural history of preserved gonads under surveillance, and developing standardized protocols for QoL assessment. Moreover, international registries and multicenter collaborations are essential to collect longitudinal data, refine risk prediction models, and optimize individualized care strategies.

## 5. Practical Guidance: When to Suspect and How to Test for NR5A1 Variants

Early recognition and targeted testing for *NR5A1* variants are essential for accurate diagnosis, personalized management, and genetic counseling in 46,XY DSD. The following points summarize the main clinical clues, testing steps, and referral pathways, consistent with current international guidance [1,46,70].

### 5.1. Clinical Scenarios in Which NR5A1 Testing Should Be Considered

- 46,XY newborn or infant with atypical genitalia (proximal hypospadias, micropenis, bifid scrotum, or bilateral undescended/dysgenetic testes) and normal 17-OHP levels (ruling out CAH); endocrine profile may show low/normal AMH and inhibin B with variable testosterone response to hCG.
- 46,XY DSD with gonadal dysgenesis features, discordant external genitalia vs. hormonal data, or persistent Müllerian structures.
- Adolescent or adult 46,XY patient with hypergonadotropic hypogonadism or primary testicular failure, particularly if childhood genital anomalies were present.
- Familial clustering or sex-limited expression: 46,XY relatives with undervirilization and/or 46,XX female relatives with primary ovarian insufficiency (POI) or infertility.
- 46,XX testicular or ovotesticular DSD.
- Associated anomalies such as splenic malformations or, rarely, adrenal dysfunction [36].
- Prenatal suspicion of ambiguous genitalia with or without family history of DSD.

### 5.2. Testing Strategy

1. Initial assessment: Perform karyotype and baseline hormonal evaluation (LH, FSH, testosterone ± hCG test, AMH, inhibin B, 17-OHP) within a multidisciplinary DSD team.
2. First-tier genetics: Order a NGS DSD multigene panel that includes *NR5A1*. Trio-based testing (patient and parents) is preferred to clarify inheritance.
3. If panel negative or phenotype complex: Escalate to WES or Whole-Genome Sequencing (WGS) to identify additional/oligogenic variants.
4. Interpretation: Apply ACMG criteria, evaluate segregation, and consider functional studies for uncertain variants. Discuss variable penetrance and the possibility of apparently unaffected carriers during counseling.
5. Follow-up and management: Positive results should trigger risk-adapted gonadal management, tumor surveillance, fertility planning, and inclusion in registries such as I-DSD for structured longitudinal care.

### 5.3. Where Testing Should Be Performed

- Genetic testing should be undertaken in accredited referral laboratories offering validated DSD multigene panels or WES.
- Prefer national or regional reference centers affiliated with Endo-ERN or DSDnet networks, ensuring standardized interpretation, confirmatory testing, and integration with multidisciplinary management.
- Cross-border clinicians may identify suitable labs via national rare disease networks or I-DSD registry partner centers. Laboratory reports should always be discussed within the multidisciplinary team to align genotype findings with clinical and surgical strategies.

## 6. Conclusions

*NR5A1*-related 46,XY DSD represents the intersection of complex genetic mechanisms, variable clinical presentations, and significant management challenges. Surgical treatment remains controversial, and recent guidelines recommend more selective indications for gonadectomy—such as the presence of streak-like dysgenetic gonads or the inability to relocate intra-abdominal gonads to a position suitable for effective surveillance. The optimal approach requires a comprehensive, individualized evaluation by a multidisciplinary team, associated with clear and continuous communication with patients and their parents/caregivers, who should be actively involved in decision-making. Beyond individualized management, early molecular diagnosis using NGS should be encouraged as a first-line investigation in all cases of unexplained 46,XY DSD, as it allows risk stratification, tailored endocrine and surgical management, and informed reproductive counseling. Systematic inclusion of *NR5A1*-related cases in international registries and multicenter studies will be crucial to establish genotype–phenotype correlations, define safe gonadal-preservation criteria, and clarify long-term fertility and psychosocial outcomes. Ultimately, the main objective is to ensure holistic care—integrating genetic, endocrine, surgical, and psychological support—through lifelong follow-up. Such an approach not only optimizes clinical outcomes but also enhances patients’ quality of life and autonomy as they transition into adulthood.

## Figures and Tables

**Table 1:** Summary of 2025 EAU classification of differences of sex development (DSD).

DSD Category	Definition/Key Features	Main Examples
46,XX DSD	Conditions with a 46,XX karyotype and virilized or ambiguous external genitalia, usually due to androgen excess during fetal life.	Congenital adrenal hyperplasia (21-hydroxylase deficiency), maternal androgen exposure, aromatase deficiency
46,XY DSD	Conditions with a 46,XY karyotype and undervirilized or ambiguous genitalia due to defects in testicular development, androgen synthesis, or androgen action.	Partial/complete gonadal dysgenesis, 5a-reductase type 2 deficiency, 17B-HSD deficiency, partial/complete androgen insensitivity syndrome
Sex chromosome DSD	Mosaic or structural abnormalities of sex chromosomes resulting in gonadal dysgenesis or mixed gonadal development. Highly variable phenotype from male to ambiguous; asymmetrical gonads; one gonad may be testicular, the other a streak gonad.	45,X/46,XY mixed gonadal dysgenesis, 45,X/46,XY Turner variants, 47,XXY (Klinefelter syndrome)
Ovotesticular DSD	Presence of both ovarian and testicular tissue, either separate or combined as an ovotestis in the same individual, previously referred to as “true hermaphroditism”	46,XX ovotesticular DSD, 46,XY ovotesticular DSD, chromosomal mosaicism (46,XX/46,XY)
Non-hormonal/Non-chromosomal DSD	Anatomical or structural abnormalities of sex development unrelated to chromosomal or hormonal dysfunction.	Cloacal exstrophy, bladder exstrophy-epispadias complex, aphallia, severe micropenis

## Data Availability

The data published in this study are available upon request from the corresponding author.

## Abbreviations

The following abbreviations are used in this manuscript:

17-OHP	17-hydroxyprogestrone
aCGH	Array Comparative Genomic Hybridization
ACTH	Adrenocorticotropic Hormone
AMH	Anti-Müllerian Hormone
ART	Assisted Reproductive Techniques
CAH	Congenital Adrenal Hyperplasia
CAIS	Complete Androgen Insensitivity Syndrome
CIS	Carcinoma In Situ
DHT	Dihydrotestosterone
DSD	Differences of Sex Development
FISH	Fluorescence In Situ Hybridization
FSH	Follicle-Stimulating Hormone
GB	Gonadoblastoma
GBY	Gonadoblastoma Locus on the Y Chromosome
GCNIS	Germ Cell Neoplasia In Situ
GCT	Germ Cell Tumor
GD	Gonadal Dysgenesis
ICSI	Intracytoplasmic Sperm Injection
IRB	Institutional Review Board
LH	Luteinizing Hormone
MRI	Magnetic Resonance Imaging
NGS	Next-Generation Sequencing
NIPS	Non-Invasive Prenatal Screening
PAIS	Partial Androgen Insensitivity Syndrome
PCR	Polymerase Chain Reaction
POI	Premature Ovarian Insufficiency
PGT-M	Preimplantation Genetic Testing for Monogenic
QoL	Quality of Life
SF-1	Steroidogenic Factor-1
SNP	Single Nucleotide Polymorphism
*TSPY*	Testis-Specific Protein Y
UGT	Undifferentiated Gonadal Tissue
US	Ultrasound
WES	Whole-Exome Sequencing
WGS	Whole-Genome Sequencing
